# The impact of loneliness and social adaptation on depressive symptoms: Behavioral and brain measures evidence from a brain health perspective

**DOI:** 10.3389/fpsyg.2023.1096178

**Published:** 2023-03-13

**Authors:** Daniel Franco-O´Byrne, Raul Gonzalez-Gomez, Juan Pablo Morales Sepúlveda, Mayte Vergara, Agustin Ibañez, David Huepe

**Affiliations:** ^1^Center for Social and Cognitive Neuroscience (CSCN), School of Psychology, Universidad Adolfo Ibáñez, Santiago de Chile, Chile; ^2^Latin American Brain Health (BrainLat), Universidad Adolfo Ibáñez, Santiago, Chile; ^3^Pontificia Universidad Católica de Chile Programa de Doctorado en Neurociencias Centro Interdisciplinario de Neurocienciass, Santiago, Chile; ^4^Facultad de Educación Psicología y Familia, Universidad Finis Terrae, Santiago, Chile; ^5^Global Brain Health Institute, University of California, San Francisco, San Francisco, CA, United States; ^6^Global Brain Health Institute, Trinity College Dublin, Dublin, Ireland; ^7^Cognitive Neuroscience Center (CNC), Universidad de San Andrés, Buenos Aires, Argentina; ^8^National Scientific and Technical Research Council (CONICET), Buenos Aires, Argentina

**Keywords:** depressive symptoms, loneliness, social adaptation, brain health, neural correlates

## Abstract

**Introduction:**

Early detection of depression is a cost-effective way to prevent adverse outcomes on brain physiology, cognition, and health. Here we propose that loneliness and social adaptation are key factors that can anticipate depressive symptoms.

**Methods:**

We analyzed data from two separate samples to evaluate the associations between loneliness, social adaptation, depressive symptoms, and their neural correlates.

**Results:**

For both samples, hierarchical regression models on self-reported data showed that loneliness and social adaptation have negative and positive effects on depressive symptoms. Moreover, social adaptation reduces the impact of loneliness on depressive symptoms. Structural connectivity analysis showed that depressive symptoms, loneliness, and social adaptation share a common neural substrate. Furthermore, functional connectivity analysis demonstrated that only social adaptation was associated with connectivity in parietal areas.

**Discussion:**

Altogether, our results suggest that loneliness is a strong risk factor for depressive symptoms while social adaptation acts as a buffer against the ill effects of loneliness. At the neuroanatomical level, loneliness and depression may affect the integrity of white matter structures known to be associated to emotion dysregulation and cognitive impairment. On the other hand, socio-adaptive processes may protect against the harmful effects of loneliness and depression. Structural and functional correlates of social adaptation could indicate a protective role through long and short-term effects, respectively. These findings may aid approaches to preserve brain health *via* social participation and adaptive social behavior.

## 1. Introduction

Depression is a potentially life-threatening disorder that affects hundreds of millions of people globally (World Health Organization, 2021; United Nations, 2022). It has well-known devastating effects on brain health, defined as the dynamic status of cognitive, emotional, and mental domains, affecting the integrity of brain structure and function (Chapman et al., 2021; Chen et al., 2022). Depressive symptomatology is often preceded by sub-clinical depression (SCD), a less severe yet debilitating expression of depressive symptoms (Zhang et al., 2022). SCD can often remain unidentified while negatively affecting brain physiology, cognition, and general health (Crockett et al., 2020). Thus, it is crucial to identify factors that can help prevent depressive symptoms and serve as early markers of permanent brain damage (Jiang et al., 2020; Zhang et al., 2022).

One early marker of depression is the experience of loneliness (Cacioppo et al., 2010; Erzen and Çikrikci, 2018), defined as emotional distress due to perceived isolation (Jeste et al., 2020). Loneliness is associated with increased depressive symptoms due to dysfunctional cognition, self-regulation (Tong et al., 2021; Steen et al., 2022), alterations in white matter (WM) structures associated with social cognition (Nakagawa et al., 2015) and cognitive control (Lam et al., 2021). However, the negative impact of loneliness can be minimized by social adaptation, defined as appropriate social functioning in line with social norms and expectations (Londono and McMillan, 2015; Neely-Prado et al., 2021; Schulte et al., 2022). Social adaptation can reduce depressive symptoms through increased social support (Berkman and Glass, 2000; Heinrichs et al., 2003; Cohen, 2020), enhanced self-esteem (Denissen et al., 2008; Joseph and De Guzman, 2021), exposure to stimulating environments (Jopp and Hertzog, 2010; Stevens et al., 2021), and enhancement of cognitive and brain reserve (Darwish et al., 2018; Zhao et al., 2020). Despite the above, little is known regarding the relationship between loneliness, social adaptation to sub-clinical depressive symptoms, and their association with brain pathophysiology.

Moreover, the experience of loneliness represents a decisive risk factor for depressive symptomatology and decreased brain health (Cacioppo et al., 2010; Erzen and Çikrikci, 2018). In a 5-year longitudinal study, it was found that loneliness predicted subsequent changes in depressive symptomatology but not vice versa (Cacioppo et al., 2010). The notion that loneliness can give rise to depressive symptoms is consistent with Beck’s cognitive model of depression (Beck, 2008; Disner et al., 2011). For example, loneliness is associated with low self-esteem and negative attributions about others and the world (Hawkley and Cacioppo, 2010; Cacioppo and Cacioppo, 2018). According to Beck (2008), such negative views about self, others, and the world are critical factors in the development and maintenance of depression (Disner et al., 2011; Jere et al., 2021; Marchetti and Pössel, 2022). In addition, negatively biased cognition and attention result in hypervigilance to threat and executive dysfunctions (Cacioppo, 2009; Hawkley and Cacioppo, 2010; Cacioppo et al., 2014), which are both considered characteristic depressive symptoms (De Zorzi et al., 2021). Loneliness also negatively effects mood *via* diminished social reward (Cacioppo et al., 2009). Diminished social reward ultimately reduces exposure to cognitively stimulating environments and participation in otherwise pleasurable activities, an essential aspect of depression (Cacioppo et al., 2014; Tan et al., 2020).

At a neurophysiological level, loneliness-related white matter (WM) alterations can help explain depressive symptomatology. A study found that loneliness (UCLA loneliness scale scores) in healthy individuals was associated with decreased WM density in crucial areas for social cognition and self-efficacy (i.e., insula, temporoparietal regions, prefrontal cortex) (Nakagawa et al., 2015). Decreased integrity in WM structures bridging these areas in depression is widely documented (Raikes et al., 2018; Vulser et al., 2018; Zhang et al., 2018; Shen et al., 2019; Liu et al., 2021). Additionally, loneliness is strongly associated with decreased connectivity of WM tracts between the insula and temporoparietal junction; these are critical nodes of the ventral attentional system (Tian et al., 2014). Attentional impairments within physiological and cognitive domains are significantly associated with depression (Keller et al., 2019). Despite the above, to our knowledge, there is no direct evidence of the relationship between brain structural correlates of loneliness and subclinical depressive symptoms.

Social adaptation can buffer against the impact of loneliness on depressive symptoms, thereby preserving brain health. Social adaptation encompasses various aspects of social functioning, such as interaction with others, interest in sociocultural activities, leisure time, and environmental mastery (Londono and McMillan, 2015; Neely-Prado et al., 2019; Schulte et al., 2022). These aspects may protect against depression-related impairments such as coping difficulties, affected cognition, negative mood, and helplessness. For example, contact with family and friends (social network) provides support resources that promote effective coping (Berkman and Glass, 2000; Cohen, 2004). Previous research shows that social support can foster brain health during depression, alleviating symptoms and enhancing self-efficacy (Panzarella et al., 2006; Alsubaie et al., 2019; Grey et al., 2020). Secondly, engagement in sociocultural activities and leisure time may protect against depressive symptoms by minimizing negative mood, preventing cognitive decline, and enhancing well-being (Fratiglioni et al., 2004; Jopp and Hertzog, 2010; Stevens et al., 2021). Lastly, manipulating the surrounding context to suit personal needs (an essential socio-adaptive process) allows control over depressive symptoms and recovery (Joseph and De Guzman, 2021).

From a neurocognitive perspective, social adaptation is sustained by social cognition and cognitive control processes. Social cognition is critical for interacting with others and navigating the social world (Frith and Frith, 2007, 2012; Gallotti et al., 2017), while cognitive control is critical to adjusting to the social context (Samadi and Sohrabi, 2016). Regarding brain structural connectivity, socio-cognitive processes, including face perception, empathy, and mentalizing (Wang et al., 2018), are associated with WM microstructure. For instance, for face perception, studies have shown that decreased microstructural integrity in the inferior longitudinal fasciculus (ILF) and inferior fronto-occipital fasciculus (IFOF) is associated with performance in face perception (Thomas et al., 2008; Tavor et al., 2014; Hodgetts et al., 2015) and emotion recognition tasks (Marstaller et al., 2016; Unger et al., 2016). Additionally, poor integrity in these tracts has been associated with low scores in empathy and mentalizing ability (Herbet et al., 2014; Parkinson and Wheatley, 2014). On the other hand, the integrity of the IFOF and IFL is also known to be linked to cognitive control processes such as attentional control (Leng et al., 2016; Sani et al., 2021), goal-directed behavior (Johnson et al., 2013; Conner et al., 2018), and emotion regulation (Liao et al., 2014; Graziano et al., 2022). Interestingly, emotion regulation processes have been consistently associated with the integrity of the arcuate fasciculus (Allan et al., 2016; David et al., 2020; Seyedmirzaei et al., 2022). For example, decreased integrity in the arcuate fasciculus is associated with aggression and anger problems in veterans (David et al., 2020). Another study showed that integrity of the arcuate fasciculus is positively correlated to performance in emotion regulation and alexithymia (inability to identify emotions) tasks, with those having better-preserved integrity showing better scores in the instruments (Seyedmirzaei et al., 2022).

Regarding brain activity, sociocognitive processes are known to be supported by parietal function (Bonini et al., 2022). Previous research shows that parietal areas are a crucial node involved in mentalizing ability and understanding the thoughts and actions of others (Lieberman, 2007; Uddin et al., 2007; Schurz et al., 2020). It is well known that activity in inferior parietal areas and the precuneus support psychological and social aspects of the mental self, such as self-referential judgments and self-appraisal (Uddin et al., 2007). Notably, an fMRI study showed that the precuneus is activated when making positive attributions about the self (Cabanis et al., 2013). This process implies a protective strategy to preserve and enhance a positive self-concept while deflecting negative feedback (Shepperd et al., 2008; Coleman, 2011). In brief, activity in the cuneus and parietal areas can support protective processes against depression. However, there is no empirical evidence about the role of parietal function in preventing and alleviating depressive symptomatology.

Despite the above, the behavioral and brain correlates by which loneliness and social adaptation explain depression are still unknown.

To cover this gap, this work aims to (1) evaluate the relationship between loneliness (socio-affective distress) and social adaptation (effective social functioning and engagement) on depressive symptoms; (2) assess the role of social adaptation against the ill effects of loneliness; and (3) examine how brain microstructural integrity and functional connectivity associated to lifestyle factors (loneliness and social adaptation) can explain depressive symptoms.

In line with previous evidence, we expect that (1) loneliness will be associated with increased severity of depressive symptoms while social adaptation will be associated with lower depressive symptoms; (2) social adaptation will decrease the magnitude of the relationship between loneliness and depressive symptoms; (3) the relationship between loneliness, social adaptation and depression is associated to brain white matter integrity and functional connectivity in areas sub serving socio-cognitive processes, cognitive control, and emotion regulation.

To test the relationship between loneliness, social adaptation and depressive symptoms, we analyzed data from an initial sample of 255 subjects who completed questionnaires tapping on each construct (loneliness, social adaptation and depressive symptoms). These initial results were validated on a second sample of 64 subjects (sample 2). Then, to assess the neurophysiological factors explaining the effects of loneliness and social adaptation on depressive symptoms, we analyzed brain scans (MRI and fMRI) belonging to sample 2.

Our findings can be of great use for interventions to enhance brain health through preventing and mitigating depression, increased engagement and social participation.

## 2. General protocol

Here we considered data from two separate samples from different studies before the pandemic. First, we considered self-report measures from a large sample (sample 1). To test the expected relationship between loneliness, social adaptation and depressive symptoms, we conducted a hierarchical multiple regression on self-report data. Then, we analyzed data from a smaller sample (sample 2) to evaluate the neurophysiological mechanism by which loneliness and social adaptation affect depressive symptoms factors; we analyzed structural (WM integrity) and FC correlates of self-report variables and their associations. Since subjects from sample 2 had also completed the same self-report instruments tapping on loneliness, social adaptation and depressive symptoms, we could replicate the hierarchical model conducted in the former sample (sample 1). All participants signed an informed consent according to the principles of the Declaration of Helsinki and received a payment as compensation for their collaboration and time. Below are the characteristics and specific procedures for each sample.

## 3. Sample 1

### 3.1. Procedure

Participants were contacted *via* telephone or social network media and invited to attend the laboratory to complete various scales tapping on depressive symptomatology, loneliness, and social adaptation.

The Universidad Diego Portales ethics committee approved every procedure of this research.

### 3.2. Participants

Two hundred and fifty-five participants between 18 and 46 years old [*M* = 31.11; SD = 8.16; female (*n* = 142)] were enrolled in this study. They had an average of 13 years of education (*M =* 13.62, SD = 3.08, range 2–23 years of education), equivalent to completed primary and secondary education, and no history of psychiatric or neurological conditions.

### 3.3. Self-report assessment

#### 3.3.1. Beck depression inventory

The Beck depression inventory (BDI) is a 21-item scale that measures depressive symptoms and their severity. Each item comprises a set of 4 statements regarding the severity of a depressive symptom. The statements range from 0 (indicating low severity) to 3 (indicating high severity). Subjects must choose the statement (or statements) that best describe their state within the last week, including the day of evaluation. The total score lies between 0 and 63 and is obtained by adding the scores of all items. Here we used the Chilean adaptation of the BDI-IA (Valdés et al., 2017). This sample showed good reliability (*α* = 0.89). For a detailed account of the instrument (see Supplementary materials).

#### 3.3.2. University of California loneliness scale

The University of California loneliness scale (UCLA) (Russell et al., 1980) is a widely used measure of the subject’s loneliness experience and satisfaction with social relationships. Here we used an abbreviated version of the UCLA (Hays and Dimatteo, 1987), comprising the following eight items: (1) “*I lack companionship*”; (2) *“I feel left out”;* (3) “*I feel isolated from others*”; (4) “*I feel alone frequently*” (5) “*There is no one I can turn to*”; (6) “*I’m unhappy with being so withdrawn”;* (7) “*People are around me but not with me*”; (8) *“I am frequently in tune with people around*.” Responses were recorded *via* a 4-point Likert scale, ranging from 1 (never) to 4 (always). The total score is obtained by inverting positive items (i.e., item 8) and summarizing the score of all items. Thus, larger scores indicate a more pronounced experience of loneliness. UCLA has shown good levels of reliability, as evidenced by an *α* coefficient of 0.89 (Russell et al., 1980). We used a shorter 8-item version that showed good reliability levels in our sample (*α* =0.84).

#### 3.3.3. Social adaptation self-administered scale

The Social adaptation self-administered scale (SASS) (Bosc et al., 1997) is a 21-item scale that explores various aspects of social functioning, such as social ties, family relationships, work and leisure time, control of the environment, and sociocultural interest. The following are examples of items tapping on each aspect: social ties (i.e., “*What value do you attach to the relationship with others?*”); family relationships (i.e., “*How frequently do you seek contact with your family members*); control of the environment (i.e., “*Do you feel able to organize your environment according to your wishes and needs?*”); engagement to work (“*How interested are you in your occupation?*”), and leisure time (i.e., “Are you interested in hobbies and leisure activities?”). Responses are recorded *via* a 4-point Likert scale (from 0 to 3). The total score ranges from 0 to 60, corresponding to minimal and maximal social adjustment. Scores between 35 and 52 are considered normal (Bosc et al., 1997). The instrument showed good reliability in our sample (*α* = 0.73).

### 3.4. Data analyses

#### 3.4.1. Self-report data

Descriptive data analysis for loneliness, social adaptation, and depressive symptomatology are displayed in Table 1. We also conducted correlation analyses, including sociodemographic data and variables of interest (see Supplementary Table S1).

**Table 1:** Performance in self-report scales for Sample 1.

	*N* = 255 (142 female, 113 male)		
	Mean	SD	Range
BDI	8.67	8.17	0–45
UCLA	16.12	4.91	1–32
SASS	42.61	6.47	21–56

BDI, Beck Depression inventory; UCLA, University of California loneliness scale; SASS, Social adaptation Self-evaluation scale; IFS, INECO Frontal Screening.

Behavioral data were analyzed with R studio (R core team, 2020). We first conducted a hierarchical multiple regression to evaluate the effects of loneliness and social adaptation on depressive symptomatology. Hierarchical multiple regression allows the evaluation of the predictive value of different groups of independent variables entered at additional steps of the analysis while controlling for variables entered in previous steps (Petrocelli, 2003). This technique is also helpful to determine (how or if) newly added variables interact with previously added variables and improve the explained variance of the model. As for the present analysis, we first specified a base model including our control variables (age, gender, and years of education). We expected that these variables would not have any significant effect on depressive symptoms (BDI score). In a subsequent step, our measure of loneliness (UCLA score) was incorporated into the variables. For the last step, we specified a model that included the social adaptation measure (SASS score).

### 3.5. Results

#### 3.5.1. Self-report measures

##### 3.5.1.1. Hierarchical multiple regression

In the first step, we entered sociodemographic control variables. Results showed that age (*β* = 0.13, *p* = 0.41), gender (*β =* −0.19, *p* = 0.16), and years of education (*β* = −0.13, *p* = 0.42) did not have significant effects on depressive symptoms (BDI scores) (see Table 2). In the second step, loneliness (UCLA score) was added to the existing predictors. Results show that after controlling for age, gender, and years of education, only loneliness (*β* = 0.63, *p* < 0.001) emerged as a significant predictor of depressive symptomatology (BDI score) [*F*(4, 50) = 10.07, *p* < 0.001]. We entered the social adaptation measure (SASS scores) in the third step. This model revealed that after controlling for non-interest variables, both loneliness (*β* = 0.48, *p* < 0.001) and social adaptation (*β* = −0.26, *p* = 0.04) significantly predicted depressive symptomatology, positively and negatively, respectively [*F*(5, 49) = 9.43, *p* < 0.001]. It is important to note that after including social adaptation, the effect of loneliness decreases by 0.14 standardized units compared to the previous step (see Table 2). Further, after comparing R^2^ between the second and third steps, the latter explains 4% more variance (and nearly 50% of the total explained variance) associated with depressive symptoms. As stated above, we confirmed that associations between the variables of interest remained significant after controlling for age, gender, and years of education.

**Table 2:** Multiple hierarchical regression output for Sample 1.

	Demographic			Loneliness			Social adaptation		
	Standardized Beta	Standardized CI	*p*	Standardized Beta	Standardized CI	*p*	Standardized Beta	Standardized CI	*p*
Age	0.13	−0.18 to 0.44	0.411	0.04	−0.21 to 0.29	0.752	0.08	−0.17 to 0.32	0.531
Gender	−0.19	−0.46 to 0.08	0.166	−0.44	−0.23 to 0.22	0.980	0.00	−0.21 to 0.22	0.988
Years of education	−0.13	−0.44 to 0.19	0.421	−0.21	−0.46 to 0.04	0.093	−0-17	−0.42 to 0.08	0.170
Loneliness				0.63	0.41–0.85	<0.001	0.48	0.22–0.74	<0.001
Social adaptation							−0.26	−0.51 to 0.01	0.044
R^2^/R^2^ adjusted	0.09/0.03			0.45/0.40			0.49/0.44		

## 4. Sample 2

Data from this sample was used to (a) replicate the hierarchical model conducted in the previous sample; (b) and assess the neurophysiological factors associated with the observed association between the interest variables.

Testing took place on two separate occasions. On day 1, participants were invited to attend the laboratory to complete various scales tapping on depressive symptomatology, loneliness, and social adaptation. On day 2, participants attended an fMRI scanning session. Images were obtained using a Siemens Avanto 1.5 T scanner with a standard head coil.

The Universidad Diego Portales ethics committee approved every procedure of this research.

### 4.1. Participants

The sample consisted of Sixty-four healthy participants between 20 and 73 years old [*M* = 36.88; SD = 13.62; female (*n* = 38)]. Subjects were recruited by accessibility from the general population. They have an average of 11 years of education (*M =* 11.1, SD = 3.09, range 2–20 years), equivalent to completed primary and incomplete secondary education. The mean educational level of this sample is reflective of the Chilean population, as noted in the latest UN Human Development Report (United Nations Development Programme, 2022). Subjects had no history of psychiatric or neurological conditions.

All participants were screened for executive dysfunction as assessed by the INECO frontal screening (Torralva et al., 2009). The mean score for our sample was 20.92 points, just above the 18-point cut-off for the Chilean population (Ihnen et al., 2013).

### 4.2. Self-report assessment

As in study 1, depressive symptoms were assessed with the Beck depression inventory (BDI; 0.83); loneliness was measured with the UCLA loneliness scale (*α* = 0.85); and Social adaptation was measured with the Social adaptation self-administered scale (SASS, *α* = 0.70).

Descriptive data analysis for our variables of interest is displayed in Table 3. We also conducted correlation analyses, including sociodemographic data and variables of interest (see Supplementary Table S2).

**Table 3:** Sample performance in self-report scales for Sample 2.

	*N* = 64 (37 female)		
	Mean	SD	Range
BDI	9.37	7.05	0–31
UCLA	8.65	5.27	0–24
SASS	42.27	7.12	27–55

### 4.3. Images data collection

Sixty-two subjects were recorded using MRI (Siemens Avanto, 1.5 T). The following parameters were applied in the T1 registers: TR = 1820 ms, TE = 3 ms, flip angle = 15 degrees, voxel size = 1 mm^3^, and the number of slices = 144. Instead, the recording parameters of resting state functional MRI (rfMRI) were TR = 3,300 ms, TE = 50 ms, flip angle = 90 degrees, voxel size = 3 × 3 × 3.75 mm^3^, number of slices = 36, number of volumes = 190. Diffusion weight images (DWI) were registered only in 60 subjects with the following parameters: TR = 5,600 ms, TE = 97 ms, flip angle = 90 degrees, in-plane resolution = 2 mm, slice thickness = 2 mm, b-value = 1,000 s/mm^2^ and 36 diffusion sampling directions.

#### 4.3.1. DWI: Preprocessing and analysis

DWI was preprocessed using the qsiprep (version 0.15.4) standard pipeline (Cieslak et al., 2021) to improve replicability and robustness.^1^ DSI Studio (v2022.08.03)^2^ was implemented for structural connectivity analysis. The data was reconstructed using q-space diffeomorphic reconstruction in the MNI coordinates (Yeh and Tseng, 2011). The output resolution and diffusion sampling length ratio were set to 2 mm and 1.25, respectively. Then, tensor metrics were calculated to quantify the mean diffusivity MD, and overall mean squared displacement of the water molecules (Hecke et al., 2016). The MD index is related to the integrity of the WM tracts. Loss of integrity is represented by an increase in MD, where MD changes can precede the alterations of the gray matter volume (Suri et al., 2014). This index has been previously correlated with neuro-plastic changes induced by behavioral tasks (Nakagawa et al., 2016; Takeuchi et al., 2017; Takeuchi and Kawashima, 2018). The rest of the DTI indexes (i.e., fractional anisotropy, axial diffusivity and radial diffusivity) are linked to damage in white matter tracts (Song et al., 2003, 2005; Budde et al., 2007; Chang et al., 2017) which is not necessarily the case in our sample of healthy subjects. Thus, the MD metric may be more sensitive to subtle neuro-plastic changes for our sample. In addition, unlike other DTI indexes, MD is independent of the number of diffusion gradients (Giannelli et al., 2010). Despite the advantages of the MD index for our objectives, we also analyzed FA, AD and RD metrics (refer to Supplementary Figures S2–S4). A deterministic fiber tracking algorithm (Yeh et al., 2013) and a nonparametric Spearman partial correlation were used to individually relate the MD index and the variables of interest, BDI, UCLA, and SASS, respectively, through diffusion MRI connectometry (Yeh et al., 2016). Additionally, to investigate the common neural substrate between these variables in the 62 subjects with MRI, a principal component analysis (PCA) was used to join their variances. To this end, the three variables were scaled, and the loading of the first dimension (PCA1) by subject was correlated with MD. All analyses were corrected for age, sex, and years of education using a multiple regression model. The seeding region comprised the whole brain, excluding the cerebellum, and 10.000 seeds were distributed randomly. False connections were removed with topology-informed pruning (Yeh et al., 2019) with four iterations; for tract selections, a *t*-score threshold of 2.5 was set. Additionally, *p* values were corrected by the false discovery (FDR), calculated through 4,000 randomized permutations to obtain the null distribution of the track length. Thus, only tracts with *p* < 0.05 FDR corrected were selected. Diffusion connectometry was based on the human structural connectome (Yeh et al., 2018) to delimit the tracts.

#### 4.3.2. rfMRI: Preprocessing and analysis

To improve replicability and robustness, the preprocessing of rfMRI was done using the fmriprep (version 22.0.2) standard pipeline (Esteban et al., 2019). In this algorithm, the head motion was estimated across full-time series, treated as a confound and expanded with the inclusion of temporal derivatives and quadratic terms (Satterthwaite et al., 2013). Then, framewise displacement was calculated as the absolute sum of relative motions (Jenkinson et al., 2002; Power et al., 2014). Motion outlier was fixed to a framewise displacement that exceeded a threshold of 0.5 mm, but none of the subjects in this study exceeded it. Furthermore, smoothing (with a gaussian kernel of 6x6x6 mm), denoising (linear regression of confounding effects of WM, cerebrospinal fluid, realignment, and scrubbing), band-pass filtering (0.001–0.009 Hz), and functional connectivity analysis were implemented in the CONN 20.b toolbox (Whitfield-Gabrieli and Nieto-Castanon, 2012) running on MATLAB R2018b. We independently associated the variables of interest (BDI, UCLA, SASS, and PCA1) with global correlation at the voxel level, which represents the average correlation coefficient between each voxel and all other voxels in the brain (Nieto-Castanon, 2020). All analyses were corrected for age, sex, and years of education using a multiple regression model. The multiple comparison problem was solved through the Gaussian random field theory (Worsley et al., 1996), which set the cluster size threshold at *p* < 0.05 FDR corrected and the voxel threshold at *p* < 0.001 uncorrected.

### 4.4. Results

#### 4.4.1. Self-report measures

##### 4.4.1.1. Hierarchical multiple regression

Hierarchical regression results are very similar to those from sample 2. In the first step, including only sociodemographic control variables results showed that age (*β* = −0.05, *p* = 0.45), gender (*β* = −0.06, *p* = 0.35), and years of education (*β* = −0.07, *p* = 0.30) did not have significant effects on depressive symptomatology (BDI scores, see Table 3). In the second step, loneliness (UCLA score) was added to de existing predictors. Results show that after controlling for non-interest variables, only loneliness (*β* = 0.50, *p* < 0.001) emerged as a significant predictor of depressive symptomatology (BDI score) [*F*(4, 249) = 21.74, *p* < 0.001]. We entered the social adaptation measure (SASS scores) in the third step. This model revealed that after controlling for non-interest variables, both loneliness (*β* = 0.31, *p* < 0.001) and social adaptation (*β* = −0.41, *p* < 0.001) significantly predicted depressive symptomatology positively and negatively, respectively, [*F*(5, 248) = 30.05, *p* < 0.001]. It is important to note that after including social adaptation, the effect of loneliness decreases by 0.19 standardized units compared to the previous step (see Table 4). Furthermore, after comparing R^2^ between the second and third steps, the latter explains 12% more variance associated with depressive symptomatology (38% explained variance). As stated above, we confirmed that associations between the variables of interest remained significant after controlling for age, gender, and years of education.

**Table 4:** Multiple hierarchical regression output for Sample 2.

	Demographic			Loneliness			Social adaptation		
	Standardized Beta	Standardized CI	*p*	Standardized Beta	Standardized CI	*p*	Standardized Beta	Standardized CI	*p*
Age	−0.05	−0.18 to 0.08	0.455	−0.06	−0.17 to 0.05	0.287	−0.05	−0.15 to 0.05	0.335
Gender	−0.06	−0.18 to 0.07	0.353	−0.05	−0.16 to 0.06	0.353	−0.05	−0.14 to 0.05	0.365
Years of education	−0.07	−0.18 to 0.06	0.309	−0.06	−0.17 to 0.05	0.291	0.03	−0.08 to 0.13	0.604
Loneliness				0.50	0.39–0.61	<0.001	0.31	0.19–0.42	<0.001
Social adaptation							−0.41	−0.52 to -0.29	<0.001
*R*^2^/*R*^2^ adjusted	0.01/−0.00			0.26/0.25			0.38/0.36		

##### 4.4.1.2. Structural connectivity (DWI)

**Figure 1:**
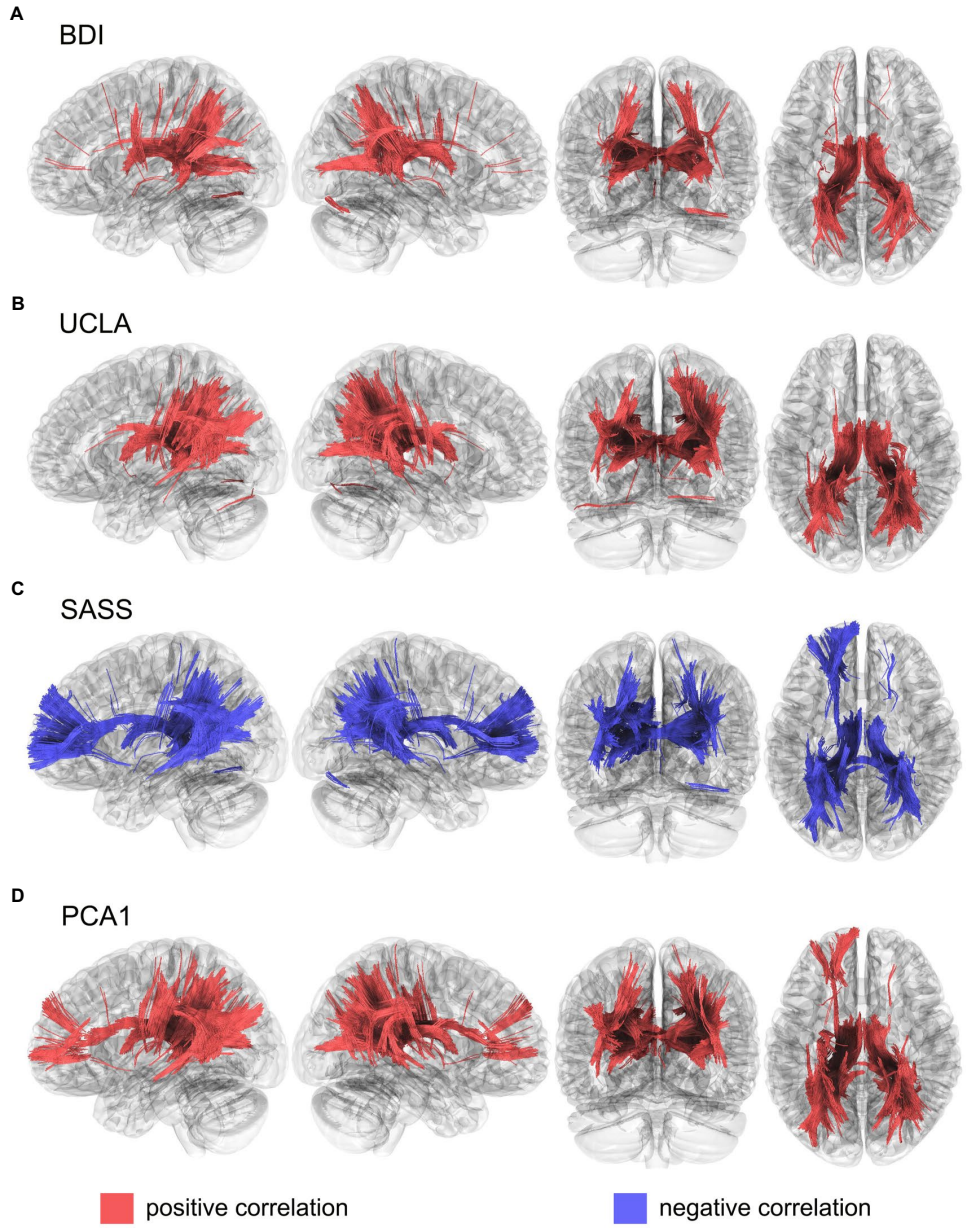
Structural connectivity correlates of **(A)**. BDI, **(B)**. UCLA, **(C)**. SASS and **(D)**. PCA1, calculated using mean diffusivity index from a diffusion tensor imaging model. Results were corrected with a *p* < 0.05 FDR. BDI, Beck depression inventory; SASS, social adaptation self-administered scale; UCLA, University of California loneliness scale; PCA1, first component from a principal component analysis that includes BDI, UCLA and SASS.

The BDI scores were positively correlated with the MD values of the fornix, corpus callosum, thalamic radiation superior and posterior, in both hemispheres, and the right segment of the inferior fronto-occipital fasciculus (Figure 1A). While UCLA scores were positively correlated with de MD index with the fornix, thalamic radiation superior and posterior, and corpus callosum in both hemispheres, and the right tracts of the inferior fronto-occipital, middle longitudinal, and corticopontine occipital tracts (Figure 1B). Instead, SASS showed a negative correlation in the tracts of the fornix, thalamic radiation superior, anterior, and posterior, corpus callosum, cortical tract posterior and inferior fronto-occipital fasciculus in both hemispheres, like so the left inferior longitudinal fasciculus, the left cingulum frontoparietal, and the left arcuate fasciculus (Figure 1C). To evaluate the common neural substrates for these three variables, we grouped their variance through the first dimension of a PCA (PCA1), which was able to explain 70.9% of the total variance (see Supplementary Figure S1). This PCA points to the different directionality of the interest variables, where BDI and UCLA were positively correlated, but SASS presented a negative correlation with both. Moreover, the PCA1 was positively correlated with the MD index of the fornix, thalamic radiation superior, anterior and posterior, corpus callosum, inferior fronto-occipital fasciculus in both hemispheres, and the left frontoparietal cingulum, left inferior longitudinal fasciculus, right arcuate fasciculus, right corticopontine occipital tract, and the right corticostriatal posterior tract (Figure 1D). Fractional anisotropy (FA), axial diffusivity (AD) and radial diffusivity (RD) showed similar patterns of results, although these appeared less pronounced (Supplementary Figures S2–S4).

##### 4.4.1.3. Functional connectivity

**Figure 2:**
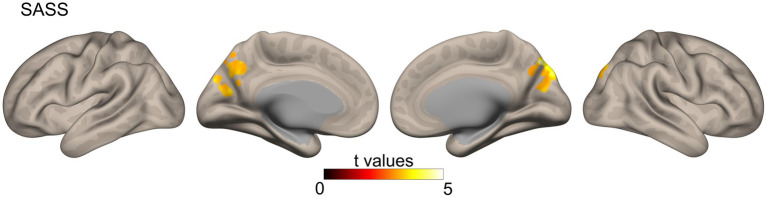
Functional connectivity correlates of SASS were calculated from a resting-state functional MRI sequence using a global correlation (connectivity between a specific voxel and the rest of the brain, measured through the average of their Pearson correlation coefficient *r*). Results were corrected with Gaussian random field theory (cluster size threshold: *p* < 0.05 FDR corrected, voxel threshold at *p* < 0.001 uncorrected). SASS, social adaptation self-administered scale.

Similar to the correlational analysis performed on structural connectivity data, we analyzed the correlations between our variables of interest (BDI, UCLA, and SASS) and the functional connectivity data. However, only the SASS scores showed significant results in areas of the precuneus, cuneus, and lateral occipital cortex for both hemispheres (Figure 2).

## 5. Discussion

Here we used data from two separate studies to evaluate the effects of loneliness and social adaptation on depressive symptoms from a behavioral and neurobiological perspective. In *sample 1*, a hierarchical regression model confirmed that loneliness (UCLA scores) negatively affected depressive symptoms (BDI scores). The model also showed that the effects of loneliness decreased after including the social adaptation measure, which positively affected depressive symptoms. In *sample 2*, we replicated our hierarchical regression model, which yielded the same pattern of assocican reveal dynamic plastic adaptations to repeated stimuluastions. We then conducted DTI analysis on imaging data from subjects in *sample 2*. Results revealed that loneliness, social adaptation, and depressive symptoms shared an overlapping WM architecture. The structures comprising this typical architecture are known to support social cognition (Herbet et al., 2014; Parkinson and Wheatley, 2014), emotion regulation (Allan et al., 2016; Vulser et al., 2018; David et al., 2020), and cognitive control (Noble et al., 2013; Herbet et al., 2018; Li et al., 2018). After conducting a functional connectivity analysis, only social adaptation was associated with functional connectivity in crucial areas for social cognition. Our findings may contribute to the understanding of the association between these variables and brain health.

Our finding that loneliness is associated with increased depressive symptoms is consistent with previous research on the long-term effect of loneliness on depression. It is known that loneliness can increase the likelihood of suffering from depressive symptomatology through low social reward, negative mood, distorted cognitive schemas, and attentional dysfunctions (Cacioppo et al., 2010; Erzen and Çikrikci, 2018). First, the diminished social reward experienced in loneliness can limit social engagement and participation in cognitively stimulating and mood-lifting activities, thereby increasing negative mood and depressive symptoms (Cacioppo et al., 2014; Tan et al., 2020). Second, it is well known that loneliness is associated with low self-esteem and negative views about others and the world (Cacioppo, 2009; Hawkley et al., 2009). Negative beliefs about the self, others and the world are key risk factors for developing and maintaining depression (Beck, 2008; Disner et al., 2011). Such a negatively biased cognition is known to underlie the attentional and executive dysfunctions typically exhibited by lonely people (Cacioppo, 2009; Hawkley and Cacioppo, 2010; Cacioppo et al., 2014) and those suffering from depressive symptoms (De Zorzi et al., 2021). Our results suggest that loneliness may represent a risk factor warning about depressive symptoms and potential progression into more severe manifestation and impairment.

On the contrary, social adaptation or appropriate social functioning can offer a range of protective factors against depressive symptoms. Specifically, interaction with others lowers emotional distress, promotes reappraisal, and facilitates overall coping (Berkman and Glass, 2000; Hostinar and Gunnar, 2015; Szkody and McKinney, 2020). Additionally, engagement in sociocultural and leisure activities may improve mood, increase behavioral activation, and prevent maladaptive cognitive patterns. Consistent with the above, we found that (1) social adaptation (adaptive social functioning) was associated with lower depressive symptoms: and (2) that social adaptation weakened the association between loneliness and depression. These results suggest that socio-adaptive behavior mitigates depressive symptoms through enhanced coping ability, improving mood, promoting stimulating activities, and preventing social isolation. These processes may also explain the protective role of social adaptation against the ill effect of loneliness. It is also important to note that the association pattern between our variables was replicated in sample 2, which strengthens the validity of our results. Therefore, our findings provide robust evidence about the role of socio-adaptative skills as a buffer against loneliness and depressive symptomatology.

Regarding our neurophysiological findings, the WM correlates of depressive symptoms reported here are in line with previous studies showing that depression is related to poor WM integrity *via* increased MD values in the fornix, thalamic radiation, corpus callosum, and inferior fronto-occipital fasciculus (Raikes et al., 2018; Vulser et al., 2018; Shen et al., 2019). In the case of loneliness, our outcomes were in line with previous evidence of the association between UCLA scores and decreased WM (high MD values) in the fornix, superior and posterior thalamic radiation, corpus callosum, right inferior fronto-occipital fasciculus, middle longitudinal, and corticopontine occipital tracts (Tian et al., 2014; Nakagawa et al., 2015; Costanzo et al., 2022). Integrity in these fibers has been previously associated with attentional control (Tian et al., 2014) and socio-cognitive (Nakagawa et al., 2015) deficits in loneliness. As for social adaptation, results showed that performance in SASS was associated with preserved WM integrity (*via* low MD values) in tracts of the fornix, thalamic radiations (superior, anterior, and posterior), corpus callosum, posterior cortical tract, inferior fronto-occipital fasciculus, left inferior longitudinal fasciculus, left frontoparietal cingulum, and the left arcuate fasciculus. These tracts are known to support cognitive control (Noble et al., 2013; Herbet et al., 2018; Li et al., 2018) and socio-cognitive abilities (Thomas et al., 2008; Herbet et al., 2014; Parkinson and Wheatley, 2014; Tavor et al., 2014; Hodgetts et al., 2015; Marstaller et al., 2016; Unger et al., 2016). Cognitive control and social cognition are core aspects of social adaptation (Huepe et al., 2011; Samadi and Sohrabi, 2016; Neely-Prado et al., 2019). This is consistent with recent conceptualizations of social adaptation as a dynamic process encompassing the accommodation own thoughts and behaviors based on social norms and expectations (Londono and McMillan, 2015; Neely-Prado et al., 2021; Schulte et al., 2022).

Social adaptation also showed associations with functional connectivity. Specifically, SASS scores were positively associated with functional connectivity in the precuneus, cuneus, and lateral occipital cortices. These areas are critical nodes in the fronto-parietal mentalizing network (Lieberman, 2007; Uddin et al., 2007; Schurz et al., 2020) and are also crucial for self-appraisal processes (Uddin et al., 2007). Moreover, the function of the precuneus has been associated with the self-serving bias (Cabanis et al., 2013), a protective strategy for enhancing self-image (Shepperd et al., 2008; Coleman, 2011). One possible interpretation for the effects of protective strategy is that it serves as a “kickstart” adaptive process that will change brain structure and behavior. It is well known that functional connectivity can reveal dynamic plastic adaptations to repeated stimulus and behavior (i.e., experience-dependent plasticity) that could later develop into more stable structural changes (Ma et al., 2011; Urner et al., 2013; Guerra-Carrillo et al., 2014; Lea-Carnall et al., 2017). For example, studies have shown that training for motor skills or visual learning is associated with the increased strength of resting state functional connectivity relative to baseline (Ma et al., 2011; Urner et al., 2013). Following this line of thought, it is reasonable to think that the protective effects of social adaptation *via* functional processes (i.e., self-serving strategy) might kickstart functional and structural changes that can prevent and even override maladaptive cognition, behavior and brain health. Moreover, just as it happens with learning, depending on the frequency of stimulation (or repetition of conduct), these initial dynamic neuro-plastic adaptations can develop into more stable structural adaptations (i.e., WM integrity above). In other words, the initial neuroplastic changes at the functional level might foment longer-term protective adaptations that can persist for months or years (Islam et al., 2020; Wilde et al., 2021).

After grouping depressive symptoms, loneliness, and social adaptation into a single component reflecting their shared variance, we found that they were associated with a common WM substrate. This common substrate included the fornix, thalamic radiations (superior, anterior, and posterior), corpus callosum, inferior fronto-occipital fasciculus, left fronto-parietal cingulum, left Inferior longitudinal fasciculus, right arcuate fasciculus, right corticopontine occipital tract, and the right corticostriatal posterior tract. We found that integrity in these tracts was negatively associated with depressive symptoms and loneliness but positively associated with social adaptation. Previous research has shown that integrity within these tracts is associated with various processes, including social cognition (Herbet et al., 2014; Marstaller et al., 2016; Unger et al., 2016; Nakajima et al., 2018; Wang et al., 2018) executive functioning (Tian et al., 2014; Li et al., 2018; Zhang et al., 2022), emotion regulation (Liao et al., 2014; Allan et al., 2016; David et al., 2020; Graziano et al., 2022; Seyedmirzaei et al., 2022), attentional control (Johnson et al., 2013; Conner et al., 2018), and goal-directed behavior (Johnson et al., 2013; Conner et al., 2018). We suggest that reduced WM integrity associated with loneliness and depressive symptoms might reflect multi-domain deficits, including emotion dysregulation, attentional impairments, poor cognitive control, and socio-cognitive abilities. This kind of deficit has been associated with depressive psychopathology. Conversely, stronger structural connectivity related to social adaptation may suggest that proficient performance in auto-regulatory (Noble et al., 2013; Herbet et al., 2018; Li et al., 2018) and socio-cognitive (Thomas et al., 2008; Herbet et al., 2014; Parkinson and Wheatley, 2014; Tavor et al., 2014; Hodgetts et al., 2015; Marstaller et al., 2016; Unger et al., 2016) may counteract the maladaptive effects of loneliness and minimize depressive symptoms.

## 6. Conclusion

Depressive symptomatology is a highly prevalent illness with profound effects on brain health (Jesulola et al., 2018; Liew, 2019; Spellman and Liston, 2020). However, most research on depression has focused on genetic markers of the illness (Jesulola et al., 2018), while social and behavioral factors have received far less attention. Loneliness and social adaptability are potential early markers for depression due to their behavioral and neural associations with SCD. Furthermore, loneliness contributes to the long-term appearance of depressive symptoms. Instead, social adaptation may modulate the relationship between loneliness and depression symptoms by reducing their effects. Thus, this socio-adaptive mechanism may involve cognitive control, strategies to augment self-image, and improved social cognition in the long and short term. These processes might benefit brain health above and beyond reducing depressive symptomatology. Brain health is a complex concept encompassing aspects of the individual (i.e., brain physiology, cognitive function, mental health, socio-cognitive abilities) and contextual level (social engagement, environmental mastery) (Chapman et al., 2021; Chen et al., 2022). Our findings can serve as a basis for socio-adaptive-based interventions to promote brain health. These interventions can prevent and help manage the severe impact of depression on cognitive and brain integrity by actively fomenting socio-adaptive factors such as social connectedness, social participation, a sense of belonging, and control of the environment. Modification of negative schemas, facilitation of interaction-rich environments, monitoring advances in social participation, and fomenting control of the environment are some ways in which “socio-adaptive” based interventions can help improve the health of individuals and communities. These interventions would be of high value, significantly, when isolation and mental health are highly compromised.

## 7. Limitations

Although we refer to the long and short-term effects of social adaptation, this interpretation should be taken with caution due to the cross-sectional nature of this study. Future research should evaluate the long-term effects of socio-adaptive processes.

We acknowledge that the range of age and education within variables could have been more homogenous. However, these variables had no significant effects on the outcome variable (or associations with predictor variables) nor affected the main effects. Further, in the case of educational level, both samples are representative of the Chilean population (United Nations Development Programme, 2022).

Here we made an interesting interpretation of the short and long-term protective effects of social adaptation *via* functional and structural neuro-plastic changes. While previous research supports these processes, these interpretations should be taken cautiously. Further research should empirically assess whether repetition of the self-enhancement protective strategy can induce initial functional connectivity neuro-plastic changes and if there is a posterior change in long-term structural changes.

## Data availability statement

The raw data supporting the conclusions of this article will be made available by the authors, without undue reservation.

## Ethics statement

The studies involving human participants were reviewed and approved by Comite de etica Universidad Diego Portales, Santiago, Chile. The patients/participants provided their written informed consent to participate in this study.

## Author contributions

DF-O and DH conceived and designed the project. DF-O, DH, and RGG contributed analysis tools and performed the analysis. DF-O, DH, RGG, JPMS, and AI conducted data analysis and interpretation. DF-O, DH, RGG, JPMS, MV, and AI participated in the drafting of the manuscript. All authors contributed to the article and approved the submitted version.

## Funding

David Huepe was supported by ANID/FONDECYT Regular (1201486, 1210195, 1210176, and 1220995). Agustin Ibañez is supported by CONICET; FONCYT-PICT (2017–1818, 2017–1820); ANID/FONDAP (15150012); ANID/PIA/ANILLOS ACT210096; and the Multi-Partner Consortium to Expand Dementia Research in Latin America (ReDLat), funded by the National Institutes of Aging of the National Institutes of Health under award number R01AG057234, and Alzheimer’s Association grant (SG-20-725707-ReDLat), the Rainwater Foundation, and the Global Brain Health Institute. The content is solely the responsibility of the authors and does not represent the official views of these institutions.

## Conflict of interest

The authors declare that the research was conducted in the absence of any commercial or financial relationships that could be construed as a potential conflict of interest.

## Publisher’s note

All claims expressed in this article are solely those of the authors and do not necessarily represent those of their affiliated organizations, or those of the publisher, the editors and the reviewers. Any product that may be evaluated in this article, or claim that may be made by its manufacturer, is not guaranteed or endorsed by the publisher.

## Footnotes

^1^ https://fmriprep.org

^2^ http://dsi-studio.labsolver.org
