# Supplementary material for: The impact of loneliness and social adaptation on depressive symptoms: Behavioral and brain measures evidence from a brain health perspective
Source: Front Psychol. 2023 Mar 13;14:1096178. doi: 10.3389/fpsyg.2023.1096178 (PMC10108715; doi:10.3389/fpsyg.2023.1096178)
Supplement: Supplementary file 1 [file Data_Sheet_1.pdf]

Supplementary materials

Beck depression inventory (BDI)

In this study, we used the Chilean adaptation of the BDI-IA (Valdés et al., 2017) which measures the intensity of depressive symptoms within the last week (Sanz, 2013). Each of the 21 items contains four statements depicting depressive symptoms at different intensities. Reports range from less severe (0) to more severe (3). Subjects are instructed to choose the statement(s) that best represent their experience within the last week. The instrument used in the present study is listed below.

En este cuestionario aparecen varios grupos de afirmaciones. Por favor lea con atención cada una. A continuación, señale cuál de las afirmaciones de cada grupo describe mejor cómo se ha sentido durante esta última semana, incluido en el día de hoy. Si dentro de un mismo grupo, hay más de una afirmación que considere aplicable a su caso, márquela también. Asegúrese de leer todas las afirmaciones dentro de cada grupo antes de efectuar la elección, (se puntuará 0-1-2-3).

A)
0. No me siento triste.
1. Me siento triste.
2. Me siento triste continuamente y no puedo dejar de estarlo.
3. Me siento tan triste o tan desgraciado que no puedo soportarlo.
B)
0. No me siento especialmente desanimado respecto al futuro.
1. Me siento desanimado respecto al futuro.
2. Siento que no tengo que esperar nada.
3. Siento que el futuro es desesperanzador y las cosas no mejorarán.
C)
0. No me siento fracasado.
1. Creo que he fracasado más que la mayoría de las personas.
2. Cuando miro hacia atrás, sólo veo fracaso tras fracaso.
3. Me siento una persona totalmente fracasada.
D)
0. Las cosas me satisfacen tanto como antes.
1. No disfruto de las cosas tanto como antes.
2. Ya no obtengo una satisfacción auténtica de las cosas.
□3 Estoy insatisfecho o aburrido de todo.
E)
0. No me siento especialmente culpable.
1. Me siento culpable en bastantes ocasiones.
2. Me siento culpable en la mayoría de las ocasiones.
3. Me siento culpable constantemente.
F)
0. No creo que esté siendo castigado.
1. Me siento como si fuese a ser castigado.
2. Espero ser castigado.
3. Siento que estoy siendo castigado.

G)
0. No estoy decepcionado de mí mismo.
1. Estoy decepcionado de mí mismo.
2. Me da vergüenza de mí mismo.
3. Me detesto.
H)
0. No me considero peor que cualquier otro.
1. Me autocritico por mis debilidades o por mis errores.
2. Continuamente me culpo por mis faltas.
3. Me culpo por todo lo malo que sucede.
I)
0. No tengo ningún pensamiento de suicidio.
1. A veces pienso en suicidarme, pero no lo cometería.
2. Desearía suicidarme.
3. Me suicidaría si tuviese la oportunidad.
J)
0. No lloro más de lo que solía llorar.
1. Ahora lloro más que antes.
2. Lloro continuamente.
3. Antes era capaz de llorar, pero ahora no puedo, incluso aunque quiera.
K)
0. No estoy más irritado de lo normal en mí.
1. Me molesto o irrito más fácilmente que antes.
2. Me siento irritado continuamente.
3. No me irrito absolutamente nada por las cosas que antes solían irritarme.
L)
0. No he perdido el interés por los demás.
1. Estoy menos interesado en los demás que antes.
2. He perdido la mayor parte de mi interés por los demás.
3. He perdido todo el interés por los demás.
M)
0. Tomo decisiones más o menos como siempre he hecho.
1. Evito tomar decisiones más que antes.
2. Tomar decisiones me resulta mucho más difícil que antes.
3. Ya me es imposible tomar decisiones.
N)
0. No creo tener peor aspecto que antes.
1. Me temo que ahora parezco más viejo o poco atractivo.
2. Creo que se han producido cambios permanentes en mi aspecto que me hacen parecer poco atractivo.
3. Creo que tengo un aspecto horrible.
O)
0. Trabajo igual que antes.
1. Me cuesta un esfuerzo extra comenzar a hacer algo.
2. Tengo que obligarme mucho para hacer algo.
3. No puedo hacer nada en absoluto.
P)
0. Duermo tan bien como siempre.
1. No duermo tan bien como antes.

2. Me despierto una o dos horas antes de lo habitual y me resulta difícil volver a dormir.
3. Me despierto varias horas antes de lo habitual y no puedo volverme a dormir.

Q)

0. No me siento más cansado de lo normal.
1. Me canso más fácilmente que antes.
2. Me canso en cuanto hago cualquier cosa.
3. Estoy demasiado cansado para hacer nada.

R)

0. Mi apetito no ha disminuido.
1. No tengo tan buen apetito como antes.
2. Ahora tengo mucho menos apetito.
3. He perdido completamente el apetito.

S)

0. Últimamente he perdido poco peso o no he perdido nada.
1. He perdido más de 2 kilos y medio.
2. He perdido más de 4 kilos.
3. He perdido más de 7 kilos.

Estoy a dieta para adelgazar SI/NO.

T)

0 No estoy preocupado por mi salud más de lo normal.
1. Estoy preocupado por problemas físicos como dolores, molestias, malestar de estómago o estreñimiento.
2. Estoy preocupado por mis problemas físicos y me resulta difícil pensar algo más.
3. Estoy tan preocupado por mis problemas físicos que soy incapaz de pensar en cualquier cosa.

U)

0. No he observado ningún cambio reciente en mi interés.
1. Estoy menos interesado por el sexo que antes.
2. Estoy mucho menos interesado por el sexo.
3. He perdido totalmente mi interés por el sexo.

Supplementary tables

Supplementary table 1

Means, standard deviations, and correlations with confidence intervals

Variable	*M*	*SD*	1	2	3	4
1. Age	31.11	8.16				
2. Years of education	13.62	3.08	-.22**			
			[-.34, -.10]			
3. BDI	8.67	8.17	-.03	-.06		
			[-.15, .09]	[-.18, .06]		
4. UCLA	16.12	4.91	.03	-.02	.50**	
			[-.10, .15]	[-.14, .10]	[.40, .59]	
5. SASS	42.61	6.47	-.04	.22**	-.55**	-.48**
			[-.16, .09]	[.10, .33]	[-.63, -.45]	[-.57, -.38]

Note. M and *SD* are used to represent mean and standard deviation, respectively. Values in square brackets indicate the 95% confidence interval for each correlation. The confidence interval is a plausible range of population correlations that could have caused the sample correlation (Cumming, 2014). * indicates $p < .05$. ** indicates $p < .01$. Abbreviations: BDI (Beck depression inventory), UCLA (University of California loneliness scale), SASS (social adaptation self-administered scale)

Supplementary table 2

Means, standard deviations, and correlations with confidence intervals

Variable	*M*	*SD*	1	2	3	4
1. Age	36.88	13.62				
2. Years of education	11.07	3.09	-.50**			
			[-.67, -.27]			
3. BDI	9.37	7.05	.17	-.21		
			[-.10, .41]	[-.45, .06]		
4. UCLA	8.65	5.27	.08	.04	.62**	
			[-.18, .33]	[-.22, .29]	[.43, .76]	
5. SASS	42.27	7.12	-.01	.07	-.53**	-.56**
			[-.26, .25]	[-.19, .32]	[-.69, -.32]	[-.71, -.36]

Note. M and *SD* are used to represent mean and standard deviation, respectively. Values in square brackets indicate the 95% confidence interval for each correlation. The confidence interval is a plausible range of population correlations that could have caused the sample correlation (Cumming, 2014). * indicates $p < .05$. ** indicates $p < .01$. Abbreviations: BDI (Beck depression inventory), UCLA (University of California loneliness scale), SASS (social adaptation self-administered scale)

Supplementary figures

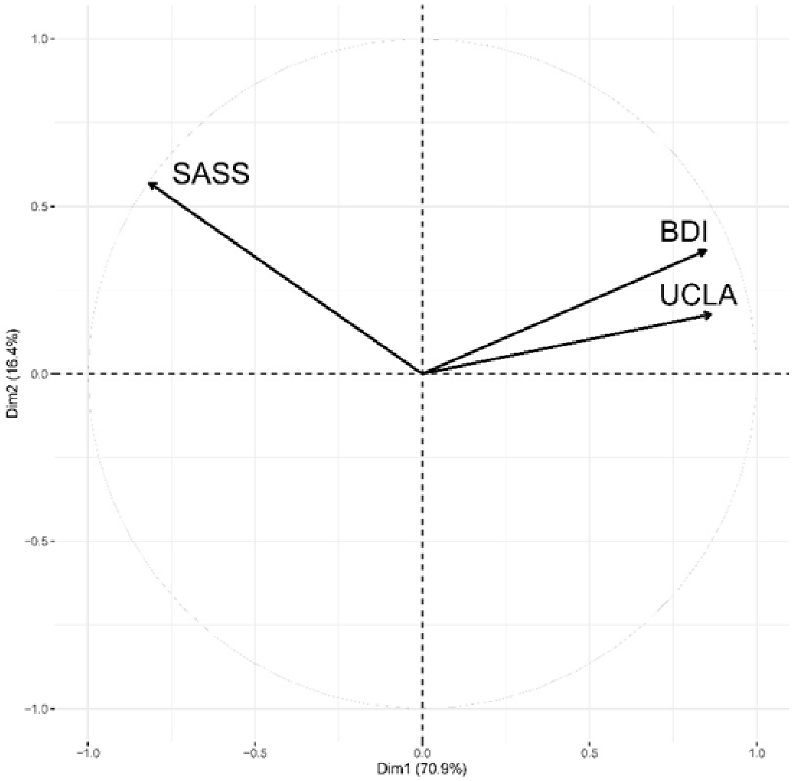

Supplementary figure 1. Spatial representation of the principal component analysis with the BDI, UCLA and SASS variables of the 62 subjects with MRI.

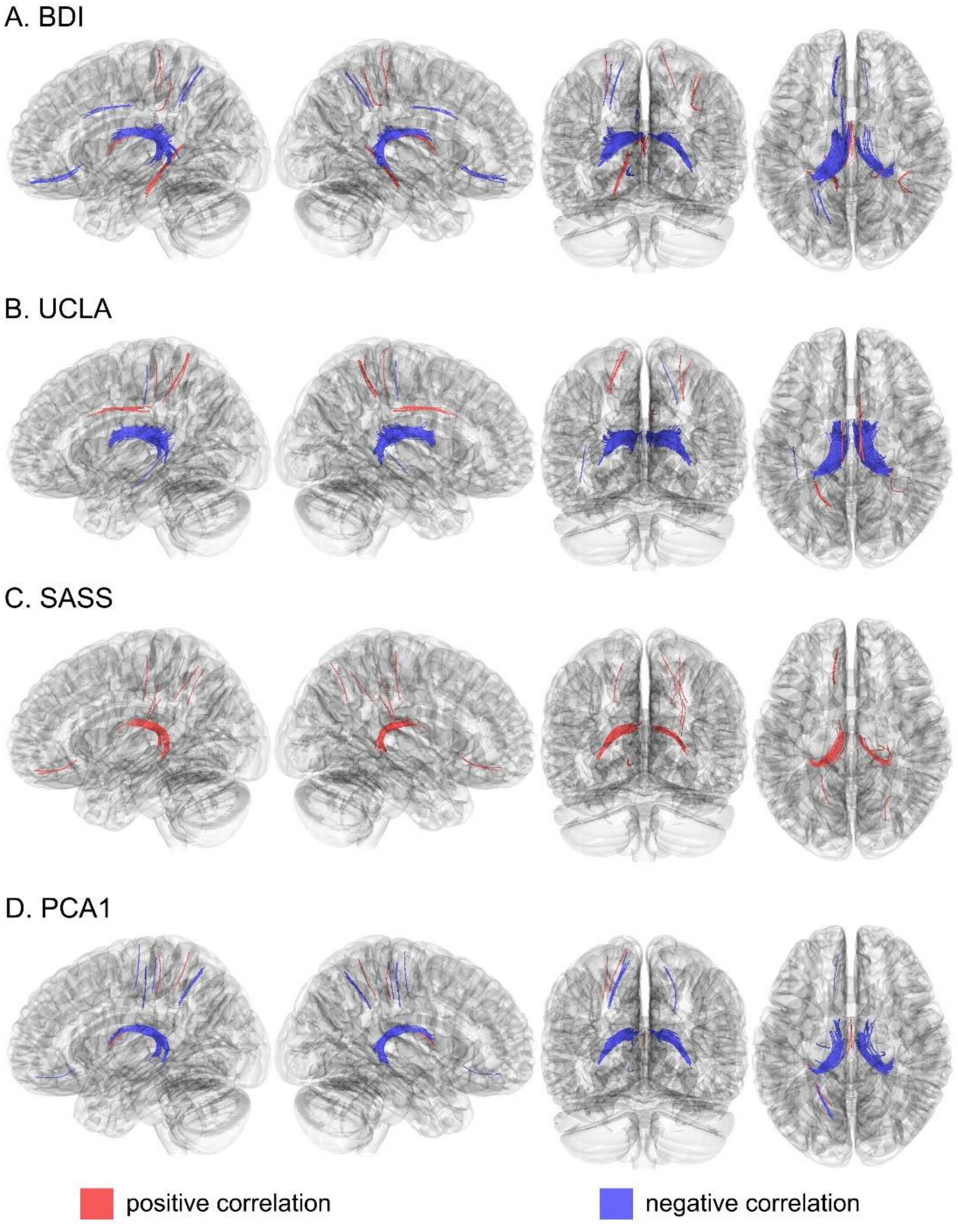

Supplementary figure 2. Structural connectivity correlates of **A.** BDI, **B.** UCLA, **C.** SASS and **D:** PCA1, calculated using fractional anisotropy index from a diffusion tensor imaging model. Results were corrected by $p < 0.05$ FDR. Abbreviations: SASS: social adaptation self-administered scale, UCLA: university of California loneliness scale, BDI: Beck depression inventory, PCA1: first component from a principal component analysis that includes BDI, UCLA and SASS scores.

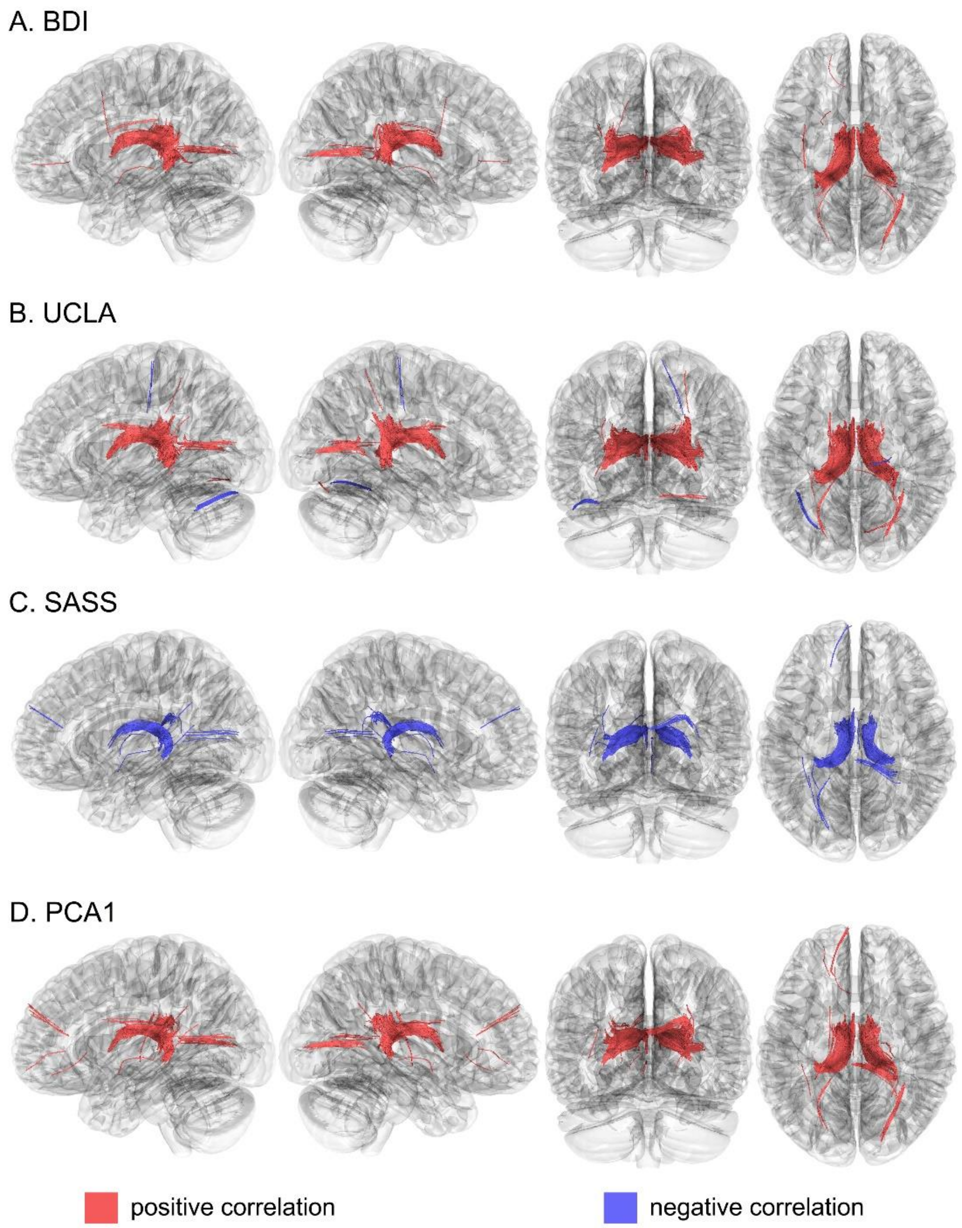

Supplementary figure 3. Structural connectivity correlates of **A.** BDI, **B.** UCLA, **C.** SASS and **D.** PCA1, calculated using axial diffusivity index from a diffusion tensor imaging model Results were corrected by $p < 0.05$ FDR. Abbreviations: BDI: Beck depression inventory, SASS: social adaptation self-administered scale, UCLA: university of California loneliness scale, PCA1: first component from a principal component analysis that includes BDI, UCLA and SASS scores.

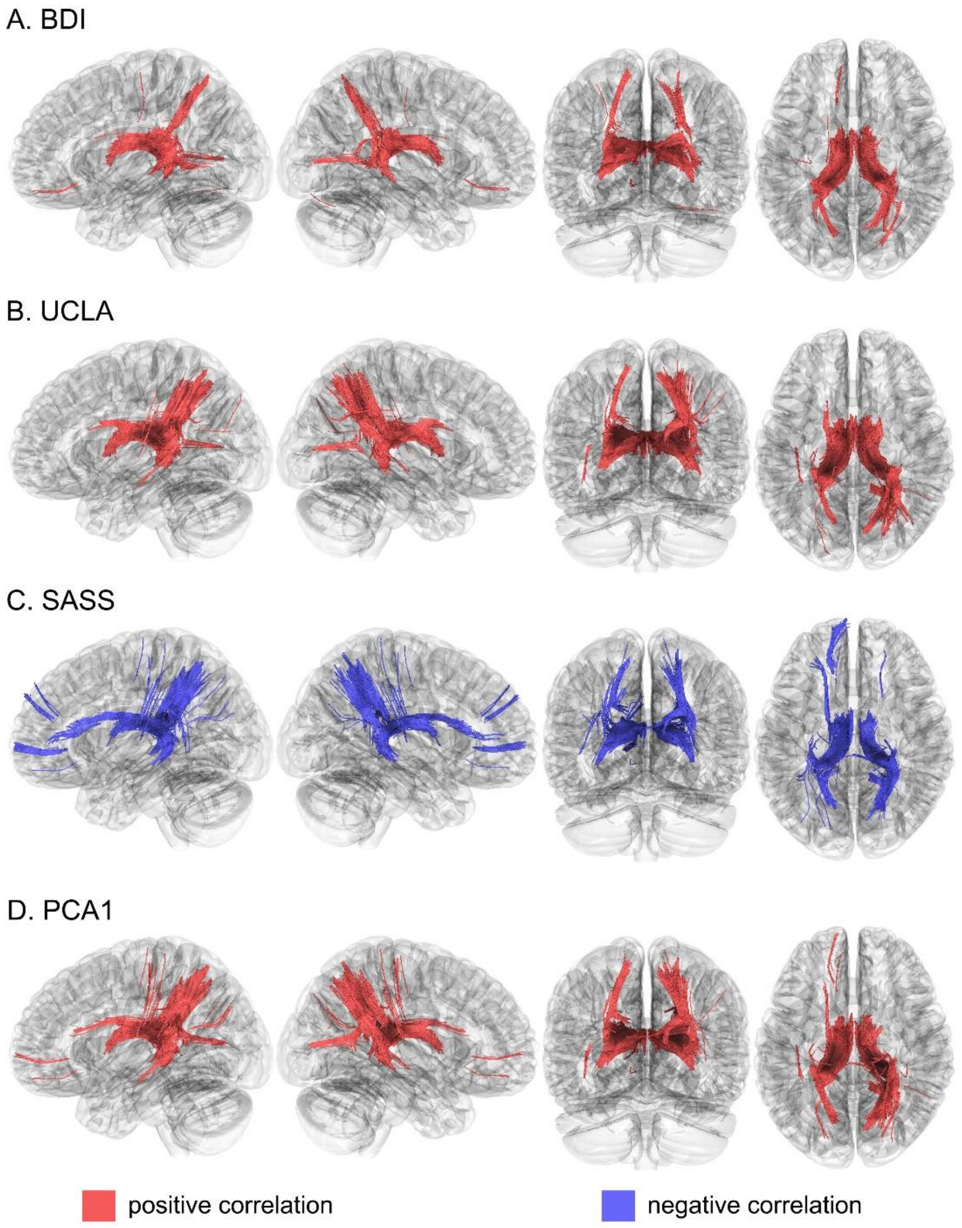

Supplementary Figure 4. Structural connectivity correlates of **A.** BDI, **B.** UCLA, **C.** SASS and **D.** PCA1, calculated using radial diffusivity index from a diffusion tensor imaging model. Results were corrected by $p < 0.05$ FDR. Abbreviations: BDI: Beck depression inventory, SASS: social adaptation self-administered scale, UCLA: university of California loneliness scale, PCA1: first component from a principal component analysis that includes BDI, UCLA and SASS scores.

References

Sanz, J. (2013). 50 años de los Inventarios de Depresión de Beck: consejos para la utilización de la adaptación española del BDI-II en la práctica clínica. *Papeles Del Psicólogo*, *34*(3), 161–168.

Valdés, C., Morales-Reyes, I., Pérez, J. C., Medellín, A., Rojas, G., & Krause, M. (2017). Propiedades psicométricas del Inventario de Depresión de Beck IA para la población chilena. *Revista Médica de Chile*, *145*(8), 1005–1012.
